# Effect of Plasma Treatment and Its Post Process Duration on Shear Bonding Strength and Antibacterial Effect of Dental Zirconia

**DOI:** 10.3390/ma11112233

**Published:** 2018-11-09

**Authors:** Chan Park, Sang-Won Park, Kwi-Dug Yun, Min-Kyung Ji, Sungwoo Kim, Yunzhi Peter Yang, Hyun-Pil Lim

**Affiliations:** 1 Department of Prosthodontics, School of Dentistry, Chonnam National University, 33 Yongbong-ro, Buk-gu, Gwangju 61186, Korea; upgradepc@hanmail.net (C.P.); psw320@naver.com (S.-W.P.); ykd@jnu.ac.kr (K.-D.Y.); asdiakonos@naver.com (M.-K.J.); 2 Department of Orthopedic Surgery, Stanford University, 300 Pasteur Drive, Edwards, R155, Stanford, CA 94305, USA; kim4@stanford.edu

**Keywords:** non-thermal atmospheric pressure plasma (NTAPP), shear bonding strength (SBS), antibacterial effect, time-dependent effect, argon gas

## Abstract

We have investigated the effect of non-thermal atmospheric pressure plasma (NTAPP) treatment and the post process time on the bonding strength and surface sterilization of dental zirconia. Presintered zirconia specimens were manufactured as discs, and then subjected to a 30-min argon treatment (Ar, 99.999%; 10 L/min) using an NTAPP device. Five post-treatment durations were evaluated: control (no treatment), P0 (immediate), P1 (24 h), P2 (48 h), and P3 (72 h). The surface characteristics, shear bonding strength (SBS) with two resin cements, and *Streptococcus mutans* biofilm formation of these plasma-treated dental zirconia were tested. Plasma did not change the roughness, and caused surface element changes and surface energy increase. Due to this increase in surface energy, SBS increased significantly (*p* < 0.05) within 48 h when RelyX^TM^ U200 was used. However, the increase of surface oxygen significantly decreased (*p* < 0.05) the SBS of Panavia F 2.0 when using plasma immediately (P0). *S. mutans* adhesion decreased significantly (*p* < 0.05) for the P0, P1, and P2 groups compared to the control. The P0 group exhibited lower biofilm thickness than the other experimental groups due to the increased hydrophilicity (*p* < 0.05). Our study suggests that there is a suitable time window for the post NTAPP treatment regarding bonding strength and antimicrobial growth persist.

## 1. Introduction

The selection criteria of dental prosthesis includes mechanical properties, aesthetics, and biological compatibility. Gold, porcelain-fused-to-metal, porcelain-fused-to-gold, and all-ceramic materials have been used in the past, but none of these materials can simultaneously satisfy the requirements for aesthetics, strength and biocompatibility. Currently, zirconia meeting these three requirements is commonly used [1,2,3]. However, even if zirconia is a good dental material, it is important to further improve its bonding strength because the detachment of between zirconia and porcelain still occurred in the oral cavity after a long time. To this end, a range of treatments have been used to improve dental materials regarding biocompatibility and bonding strength, etc. [4,5,6]. For example, surface treatment of the prosthesis or teeth such as increased surface roughness can improve the bonding strength [7,8,9,10,11], but, a rougher surface can encourage the adhesion of bacteria [12,13].

As such, there is a great need for surface treatment methods such as plasma treatment [14,15,16,17,18,19,20,21,22] that improve the bonding strength without changing the physical properties, such as surface roughness. In particular, non-thermal atmospheric pressure plasma (NTAPP) is one possible means of optimizing the Y-TZP surface chemistry to improve bond strength while producing an antibacterial effect [23]. Especially in environmental engineering, it is known that the ozone generation due to plasma causes biological degradation for the sterilization effect [24,25,26,27]. The increase in surface energy resulting from the application of NTAPP increases the interaction between different materials for increasing bonding strength without changing the surface roughness [28]. 

Various studies have investigated differences of the effects of plasma treatment depending on the surface of the specimen [29,30], the plasma irradiation time [31], and the plasma gas [32]. However, the relationship between the plasma effect and the length of time following irradiation remains unclear. Therefore, an investigation of the persistence of the effects of plasma irradiation during storage in atmosphere is warranted. 

The present study investigated the differences in the bonding strengths of two resin cements, and the antibacterial activity toward *S. mutans* after plasma-treated zirconia specimens had been stored for different intervals. We also compared the effects of the plasma treatment on different resin cements. The null hypothesis was that storage time does not affect bonding strength or antimicrobial activity.

## 2. Materials and Methods

### 2.1. Sample Preparation

Presintered zirconia specimens (Zirmon^®^, Kuwotech, Gwangju, Korea) were prepared by fully sintering the pre-zirconia specimens at 1450 °C for 2 h in a high temperature furnace (KaVoTherm, KaVo Dental GmbH, Biberach, Germany). The final specimens (diameter: 15 mm, height: 2.5 mm) were wet-polished using abrasive papers (#600 silicon carbide [SiC] followed by #800 SiC). The specimens were then washed in acetone, alcohol, and distilled water (20 min each) in an ultrasonic bath.

### 2.2. NTAPP

**Figure 1:**
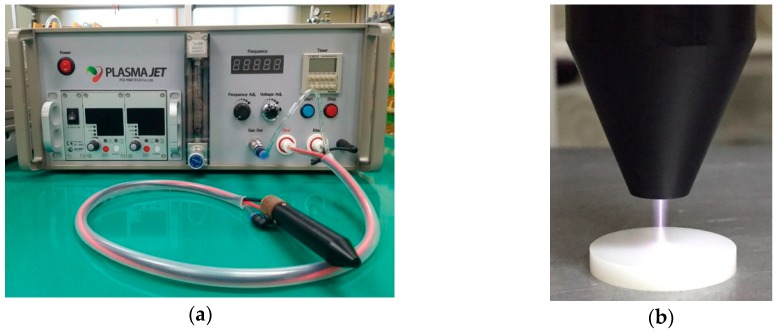
Non-thermal atmospheric plasma generator: (**a**) Frontal view; (**b**) Plasma treatment of a zirconia specimen.

Argon gas (Ar_2_, 99.999%) was used for plasma generation. The gas was injected into the atmospheric pressure plasma apparatus (Plasma Jet, Polybiotech Co., Ltd., Gwangju, Korea) at a flow rate of 10 L/min. Plasma was generated by applying 10 W of power. During the plasma treatment, the flame was held approximately 5 mm from the specimen. Based on the results of a previous study [33] and preliminary experiments, the treatment was performed for 30 min (Figure 1). The specimens were randomly divided into five groups (Table 1).

### 2.3. Resin Cement

Adhesive resin cement (Panavia F2.0; Kuraray Medical Inc., Osaka, Japan) containing methacryloyloxydecyl dihydrogen phosphate (MDP), and self-adhesive resin cement (RelyX^TM^ U200; 3M ESPE, St Paul, MN, USA) containing a self-etching primer were used. The composition of each is summarized in Table 2.

### 2.4. Surface Analysis

#### 2.4.1. Surface Roughness

Atomic force microscopy (AFM) was used to investigate the change in surface roughness after plasma irradiation. A PSIA XE-100 scanning electron microscope (Park Systems, Suwon, Korea) was used. The average surface roughness (Ra), measured at the center of one specimen (45 × 45 µm) of each experimental group, was calculated and compared. The Ra values for three specimens were measured for each test group and the mean value was calculated.

#### 2.4.2. X-ray Diffraction

An X’Pert PRO Multi-Purpose X-ray Diffractometer (PANalytical, Almelo, The Netherlands) was used to investigate the structural changes in the zirconia crystal phase after plasma irradiation. Radiation of CuKα = 1.54 Å at 60 kV and 55 mA was applied at 2 degrees/min for 2θ, ranging from 20 to 90 degrees.

#### 2.4.3. Energy-Dispersive X-ray Spectroscopy and X-ray Photoelectron Spectroscopy

After plasma irradiation, the chemical composition and bonding state of the surface were investigated using energy-dispersive X-ray spectroscopy (EDS; S-4700, Hitachi, Tokyo, Japan) and X-ray photoelectron spectroscopy (XPS; VG Multilab 2000, Thermo Scientific, Waltham, MA, USA). The EDS results were used to calculate the mass ratio and the element weights of the carbon (C), oxygen (O), zirconium (Zr), and argon (Ar). The XPS results provided the normalized values for the area. They were expressed as quantitative ratios; the peak of each element was calculated using carbon (C1s) and oxygen (O1s).

#### 2.4.4. Apparent Contact Angle

An apparent contact angle goniometer (Phoenix 300; SEO, Gyeonggi-do, Korea) and Image XP software (SEO) were used to evaluate the change in hydrophilic surface properties by measuring the apparent contact angle after plasma treatment [34]. Distilled water (4 μL) was dropped vertically onto the zirconia specimen. The angle between the surface and the liquid was measured after 10 s using a apparent contact angle meter. Three specimen apparent contact angles were obtained for each test group, and the mean value was calculated.

### 2.5. SBS Testing

One hundred specimens were used to measure the SBS between each resin cement and the plasma-treated zirconia specimens. Round blocks of composite resin (Filtek^TM^ Z250, 3M ESPE, Maplewood, MN, USA) were made using a plastic mold (diameter: 6 mm, height: 3 mm), and were polymerized using an Elipar Freelight 2-light-emitting diode (LED) light-curing unit (3M ESPE). The composite resin blocks were each bonded to a zirconia surface using resin cement and the vertical application of 10 N of pressure. The Panavia F2.0 was polymerized by applying the LED light-curing unit to each specimen from four directions, for 30 s in each direction, according to the manufacturer’s instructions. The RelyX^TM^ U200 resin self-polymerized. The maximum load (P) was determined to be the point at which the composite resin block separated from the zirconia by the application of a load at a speed of 0.5 mm/min after fixing the specimen in the jig of an RB Model 301 universal testing machine (Unitech MTM, Seoul, Korea). The fracture pattern was observed using scanning electron microscopy (SEM) after coating the fracture surface with gold-palladium alloy. Three types of fracture pattern were observed: adhesive failure (the resin cement was completely removed), cohesive failure (the fracture occurred within the resin cement), and mixed failure (both fracture patterns could be observed). The distribution of the fracture patterns was investigated according to the experimental group and the type of resin cement.

### 2.6. S. mutans Biofilm Formation

#### 2.6.1. Preparation

*S. mutans* was selected for this experiment because it is distributed throughout the oral cavity and is responsible for early bacterial membrane formation. *S. mutans* KCOM 2804 was obtained from the Korean Collection for Oral Microbiology (KCOM, Gwangju, Korea). Biofilm formation was evaluated using three specimens in each experimental group. Each specimen was transferred to a 24-well plate that had been disinfected with ethylene oxide gas. Artificial saliva was dispensed onto the specimen and was slowly stirred for 2 h in a 37 °C incubator to coat the specimen. Excess artificial saliva was removed and each specimen was naturally dried for 15 min. *S. mutans* was inoculated onto each dried specimen at a concentration of 1.5 × 10^7^ colony forming units (CFU)/mL and cultured under aerobic conditions for 24 h.

#### 2.6.2. Confocal Laser Microscopy Evaluation of Biofilm Formation

The culture solution was removed and unattached microorganisms were discarded by washing twice with phosphate-buffered saline solution. To evaluate the biofilm formation, 200 µL of fluorescent reagent (green fluorescent nucleic acid stain, SYTO 9^®^, Molecular Probes Europe BV, Leiden, the Netherlands) was used. The stain was applied to each specimen (1.5 µL/mL distilled water), the well plate was covered with aluminum foil to prevent light from entering, and the reaction was carried out at room temperature for 15 min. The remaining fluorescent reagent was carefully washed away with phosphate-buffered saline and the degree of biofilm formation was observed by confocal laser scanning microscopy (Leica TCS SP5 AOBS/tandem; Leica, Mannheim, Germany). The biofilm thickness was measured using the LAS AF software (Leica Microsystems, Bensheim, Germany). The thickness was determined at three positions per sample, yielding a total of nine biofilm formation thickness measurements for each group.

### 2.7. Statistical Analysis

All statistical analyses were performed using SPSS Statistics for Windows, Version 21.0 (IBM Korea Inc., Seoul, Korea). SBS and biofilm thickness were evaluated with a one-way analysis of variance (ANOVA), and post-hoc comparisons were made using Tukey’s test. A *p*-value of < 0.05 was considered significant.

## 3. Results

### 3.1. Surface Analysis

#### 3.1.1. Surface Roughness

**Figure 2:**
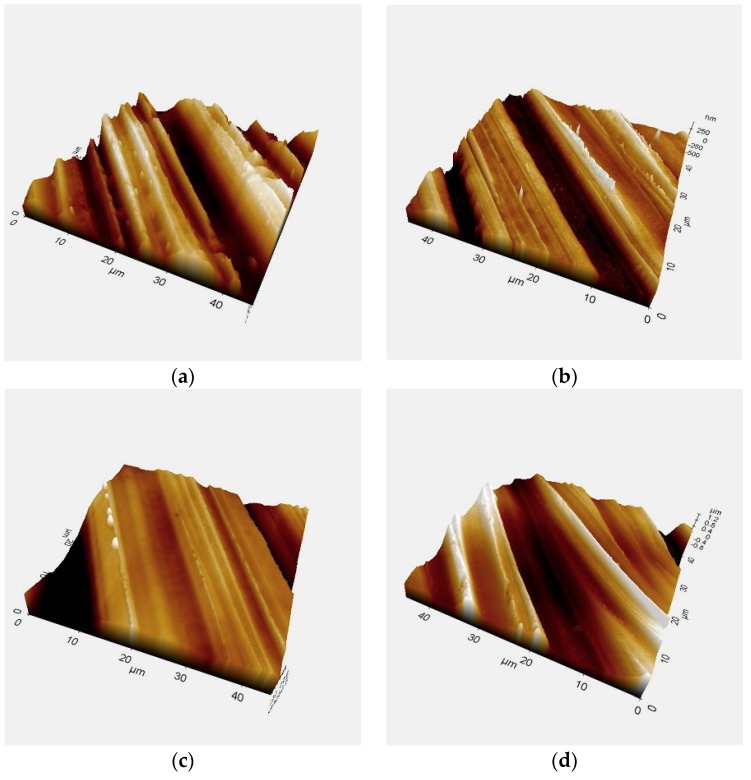

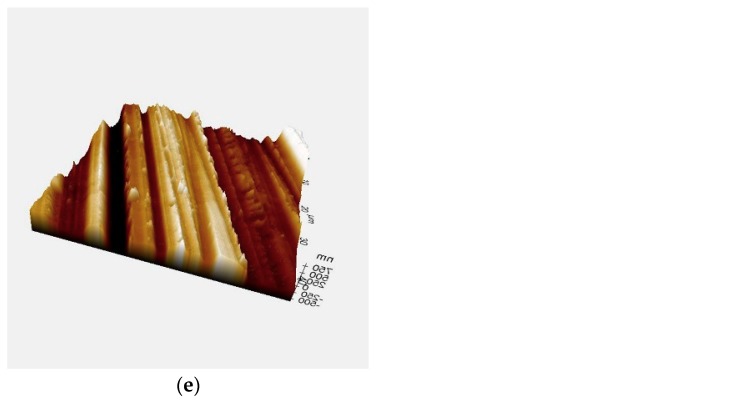
Surface after plasma treatment (45-µm × 45-µm magnification). (**a**) Control (no treatment), (**b**) P0 group (immediately after treatment), (**c**) P1 group (24 h after treatment), (**d**) P2 group (48 h after treatment), (**e**) P3 group (72 h after treatment).

The surface morphology of the zirconia observed by AFM was the same for all experimental groups regardless of plasma treatment (Figure 2). The Ra values were 67.5 ± 17.45 nm for the control group, 57.66 ± 8.06 nm for the P0 group, 38.61 ± 4.56 nm for the P1 group, 53.6 ± 18.71 nm for the P2 group, and 54.15 ± 11.13 nm for the P3 group. There were no significant differences in surface roughness after the plasma application (*p* > 0.05).

#### 3.1.2. XRD

**Figure 3:**
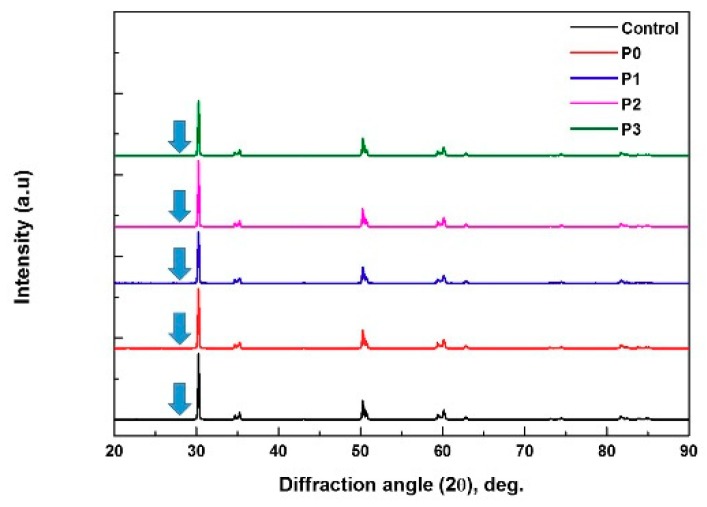
X-ray diffraction patterns of all zirconia groups after plasma treatment. Monoclinic phases were not seen in every experimental group (blue arrow).

A graph comparing the phase shifts developed from the XRD data after plasma treatment is presented in Figure 3. The application of plasma did not affect the monoclinic phase in any experimental group.

#### 3.1.3. EDS and XPS

**Figure 4:**
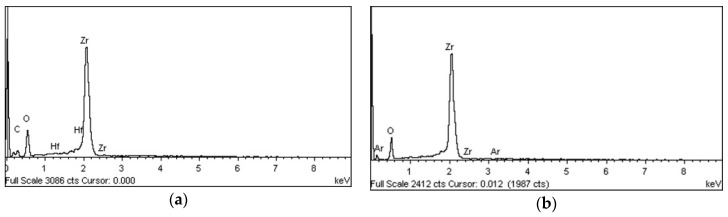

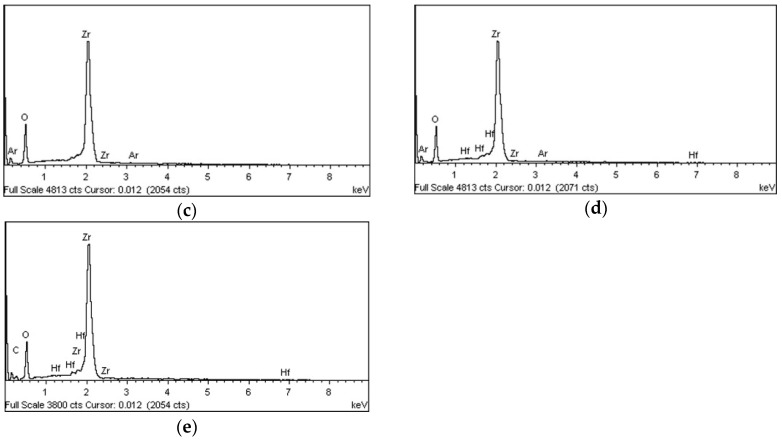
Energy-dispersive X-ray spectroscopy elemental spectrum after plasma treatment. An attempt was made to detect carbon (C), oxygen (O), argon (Ar), and zirconium (Zr). (**a**) Control (no treatment), (**b**) P0 group (immediately after treatment), (**c**) P1 group (24 h after treatment), (**d**) P2 group (48 h after treatment), (**e**) P3 group (72 h after treatment).

An analysis of the change in the elements on the zirconia surface after plasma irradiation did not reveal any argon on the surface of the control group; trace amounts of argon were detected in the P0 (0.29%), P1 (0.02%), and P2 (0.09%) groups, but not in the P3 group. No argon was detected in the control or P3 groups (Figure 4, Table 3).

Carbon (1s) and oxygen (1s) detected in the zirconia specimens after plasma treatment were present in different ratios, depending on the group (Table 4). The C:O ratio before plasma irradiation was 1.60. The oxygen content increased rapidly in the P0 group immediately after the plasma irradiation, resulting in a C:O ratio of 0.31. Each group exhibited a gradual recovery over time (0.44 for the P1 group, 0.40 for the P2 group, and 0.59 for the P3 group). However, the values differed from the control. 

#### 3.1.4. Apparent Contact Angle

**Figure 5:**
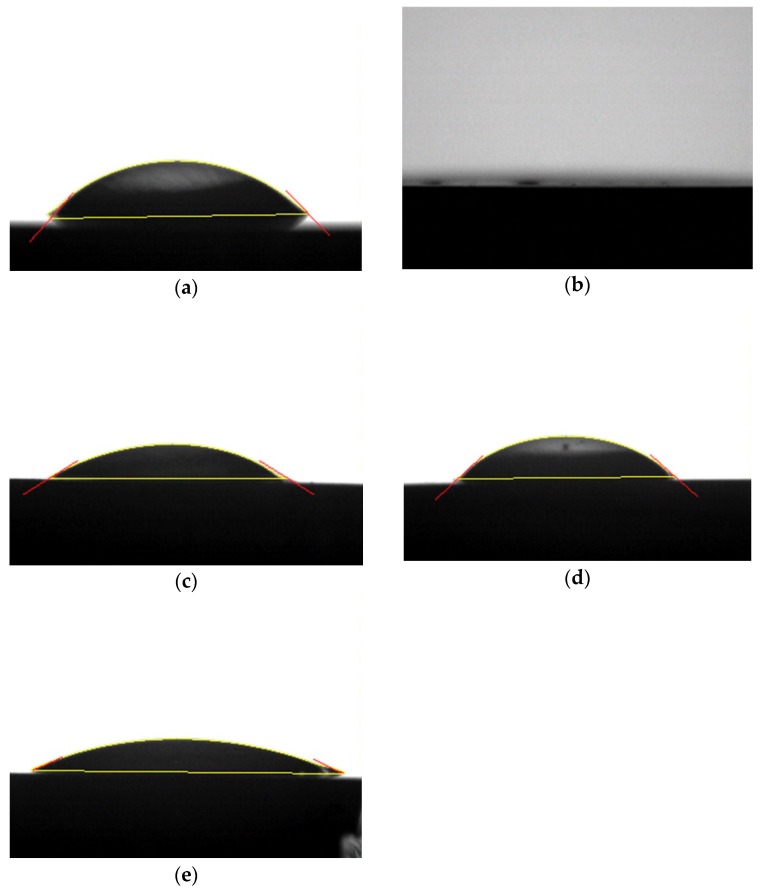
Apparent contact angle measurement of a drop of distilled water after plasma treatment. (**a**) Control (no treatment), (**b**) P0 group (immediately after treatment), (**c**) P1 group (24 h after treatment), (**d**) P2 group (48 h after treatment), (**e**) P3 group (72 h after treatment).

Before the plasma treatment, it showed a characteristic of hydrophobic surface (Figure 5a). However, when an attempt was made to measure the apparent contact angle immediately after atmospheric plasma treatment of the zirconia surface, the distilled water was found to have completely spread over the surface (Figure 5b). In other words, significant changes were observed between the surface group immediately after plasma treatment and the other groups (Figure 5).

### 3.2. SBS Test

#### 3.2.1. SBS Test with Panavia F2.0

**Figure 6:**
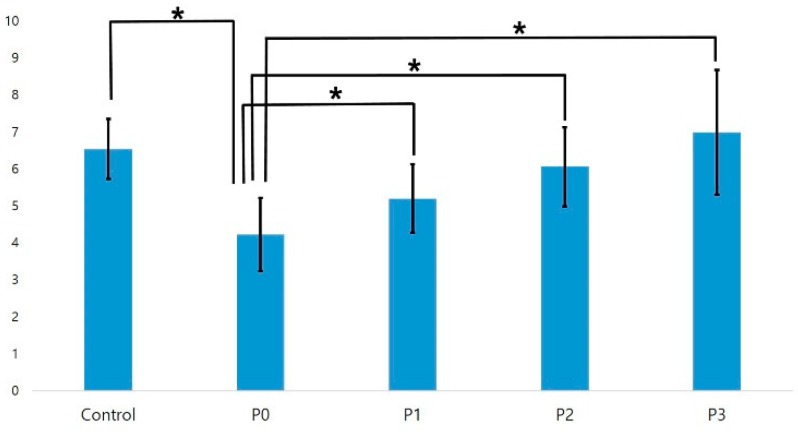
Results of a one-way analysis of variance of the shear bond strength of Panavia F2.0 (*n* = 10). An * denotes a significant difference (*p* < 0.05).

The SBS values of the zirconia and composite resin blocks were determined using Panavia F2.0. The value for the P0 group was significantly lower than that for the control group (*p* < 0.05). The bonding strength gradually increased over time, and there was no significant difference in the bond strength between the P1, P2, or P3 groups and the control group (*p* > 0.05; Figure 6). A fracture analysis showed that all experimental groups presented mixed failures (Table 5).

#### 3.2.2. SBS Test with RelyXTM U200

**Figure 7:**
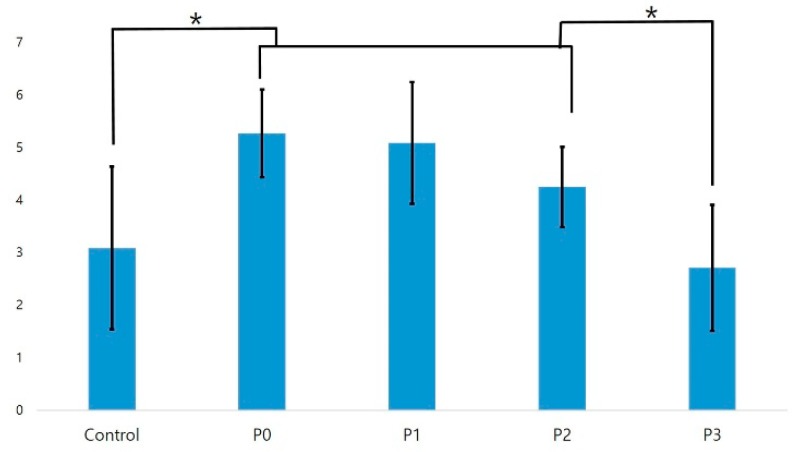
Results of a one-way analysis of variance of the shear bond strength of RelyXTM U200 (*n* = 10). An * denotes a significant difference (*p* < 0.05).

The SBS of the zirconia and composite resin blocks using RelyX^TM^ U200 was significantly higher for the P0, P1, and P2 groups than for the control group; there was no significant difference between the P3 and control groups (Figure 7). In the analysis of the fracture sections, the control and P3 groups with low bonding strengths showed adhesive failure, with the exception of one specimen in the control group. All specimens of the P0, P1, and P2 groups, which exhibited high bonding strengths after plasma treatment, exhibited mixed failure patterns. Table 5 summarizes the SBS values and failure patterns of the zirconia and composite resin blocks fabricated using the two resin cements.

### 3.3. Biofilm Formation

**Figure 8:**
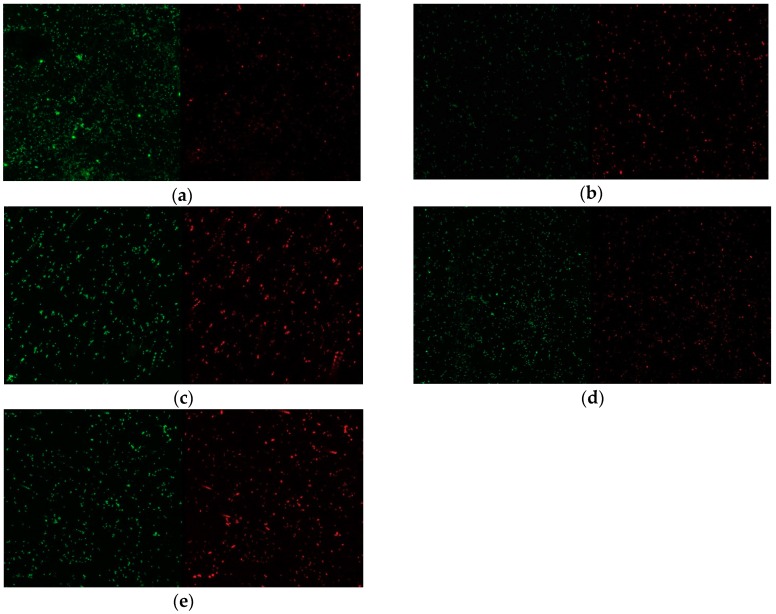
Cell viability images of Streptococcus mutans. Green fluorescence indicates viable cells; red fluorescence indicates dead cells. The number of live cells in the P0 group was reduced compared to the control group. (**a**) Control (no treatment), (**b**) P0 group (immediately after treatment), (**c**) P1 group (24 h after treatment), (**d**) P2 group (48 h after treatment), (**e**) P3 group (72 h after treatment).

**Figure 9:**
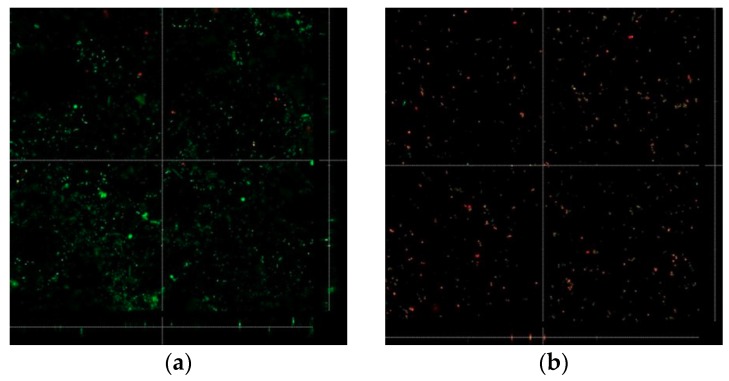

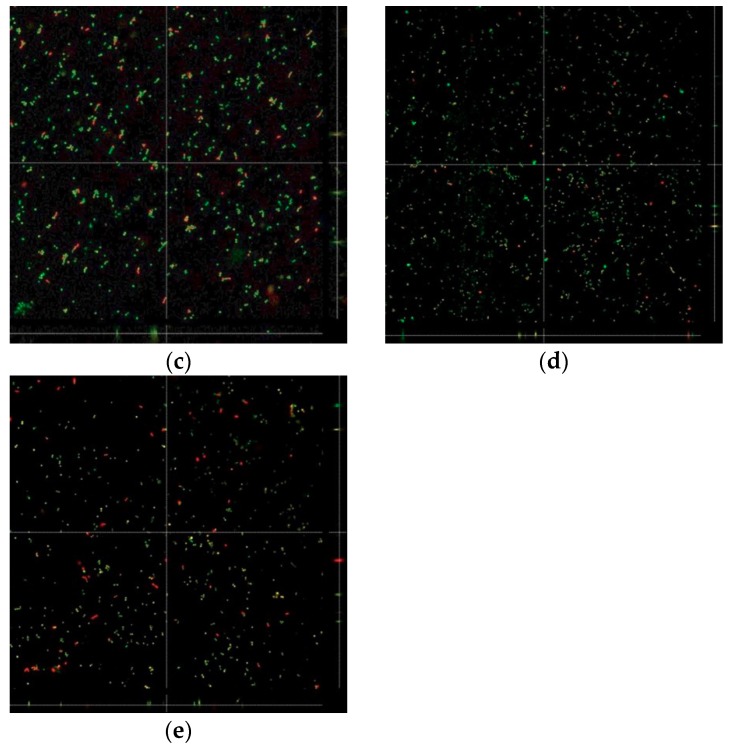
Biofilm thickness of Streptococcus mutans. Green fluorescence indicates viable cells; red fluorescence indicates dead cells. The thickness of the biofilm in the photograph can be determined from the right and bottom lines. (**a**) Control (no treatment), (**b**) P0 group (immediately after treatment), (**c**) P1 group (24 h after treatment), (**d**) P2 group (48 h after treatment), (**e**) P3 group (72 h after treatment).

**Figure 10:**
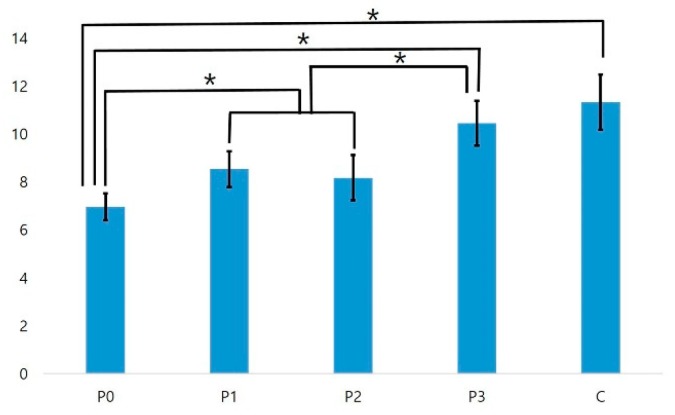
Biofilm thickness of *Streptococcus mutans* in micrometers (*n* = 3). An * denotes a significant difference (*p* < 0.05).

Many of the active bacteria, stained green in each group, died as a result of plasma treatment or the antibacterial properties of the zirconia itself (red, Figure 8). The changes in biofilm thickness are shown in Figure 9 and Figure 10. The film thickness was 6.96 ± 0.56 µm for the P0 group, 8.54 ± 0.41 µm for the P1 group, and 8.18 ± 0.95 µm for the P2 group, while the control group thickness was 11.34 ± 1.14 µm. The P0, P1, and P2 experimental groups all exhibited a significant decrease in biofilm thickness compared to the control group (all, *p* < 0.05). The P0 group also exhibited a statistically thinner film relative to the P1 and P2 groups (*p* < 0.05). The film thickness of the P3 group was 10.46 ± 1.14 µm, which was not significantly different from that of the control group (*p* > 0.05).

## 4. Discussion

In the present study, the persistence of the effects of non-thermal plasma treatment of zirconia on SBS and *S. mutans* activity was measured at various time points under atmospheric conditions. The RelyX^TM^ U200 SBS had increased at 48 h after plasma treatment. When using Panavia F2.0, however, the SBS decreased relative to that of the control group immediately after plasma treatment. Therefore, the first part of the null hypothesis, that there would be no difference in bonding strength, was rejected. Evaluation of the *S. mutans* biofilm indicated a reduction in the biofilm thickness even at 48 h after plasma treatment. Thus, the second part of the null hypothesis, that there would be no significant difference in antimicrobial activity, was also rejected.

Among the conditions affecting plasma generation, temperature and pressure both directly influence the feasibility of plasma generation in a dental clinic [21,22]. Plasma generated from the gas at room temperature and atmospheric pressure can be applied to both the teeth and soft tissues in the oral cavity. In the present study, a plasma generator was used to maintain the temperature at approximately 30 °C, while consuming very little power.

In general, the methods used to increase the bonding strength of dental ceramics include changing the texture properties of the surface through the application of hydrofluoric acid, as well as inducing chemical changes using silane coupling agents [35]. However, increasing the bond strength of zirconia is difficult, because zirconia does not contain silica, and it does not corrode [36]. In addition, since zirconia is hydrophobic and has a low surface density of hydroxyl groups, it is even more difficult to increase the bonding strength of zirconia [28]. A method has been introduced whereby the mechanical bonding strength is enhanced by increasing the surface roughness of the zirconia by spraying it with alumina particles [37,38]. The presence of the alumina particles produces cracks and a phase shift in the zirconia surface. In addition, a decrease in bonding strength due to drop-out of the remaining particles has been described [38]. Various methods to overcome these limitations, including plasma treatment, have been studied [39,40,41], and the plasma treatment approach has been verified [21,22]. An increase in the number of oxygen atoms and a decrease in the number of carbon atoms after plasma treatment have been confirmed by XPS analysis [32]; the present study results were consistent with those results. It has been reported that plasma treatment accelerates the increase in surface hydroxylation [42]. Other studies have suggested that plasma treatment destroys the C-C and C-H bonds, and removes the contaminants [29]. This was verified by the increased amount of carbon identified in the XPS analysis. Such changes in the surface properties result in increased hydrophilicity, which can lead to improved wetting of the primer or resin cement [42,43,44]. In addition, the results of the XRD analysis confirmed that non-thermal atmospheric plasma can increase the bonding strength without affecting the zirconia phase.

The strength of bonding between the zirconia and RelyX^TM^ U200 resin cement was increased significantly by plasma treatment. This may be due to the hydrophilicity, wettability, and energy increase of the surface, as previously described [45]. However, the strength of bonding to Panavia F2.0 resin cement decreased immediately after plasma treatment, which was inconsistent with previous findings [20]. The reduction in bonding strength is thought to be caused by the MDP content of the Panavia F2.0. Resin cement containing MDP can attain a high bonding strength via reaction with the hydroxyl groups on the zirconia surface [45,46,47,48,49]. However, when MDP-containing resin comes into contact with oxygen, the oxygen reacts with the radical polymerization catalyst, preventing the resin monomer molecules from binding to each other [50]. To reduce this binding interference in clinical situations, the surface is blocked from contact with air until the material is cured. The EDS and XPS analyses showed that when zirconia is treated with non-thermal plasma, the amount of oxygen on the surface increases instantly, which interferes with polymerization of the MDP-containing resin.

In the present study, we investigated the SBS immediately after plasma treatment, and assessed the influence of the elapsed time on the SBS after atmospheric storage. Therefore, this phenomenon can be explained by the “hydrophobic recovery theory” mentioned in the previous paper [51]. Atmospheric storage for 24 h can significantly reduce bonding strength values [46]. However, we found that RelyX^TM^ U200 showed statistically significantly higher bonding strength values, even at 48 h after plasma irradiation. Thus, high surface hydrophilicity, as represented by the low apparent contact angle, is not the fundamental cause of increased bonding strength. In other words, SBS and apparent contact angle do not show exact correlation. This is because it is difficult to accurately measure the contact angle hysteresis phenomena and it is difficult to clearly distinguish between surface roughness, chemical heterogeneity, and anisotropy of surface [52,53,54]. In addition, there are complex causes such as the influence of argon gas and generation of ozone [24,25,26,27]. Therefore, analysis of the surface of the plasma by the contact angle is limited due to this reason. However, this paper has shown a definite hydrophilic effect image immediately after plasma treatment (Figure 5b), and other previous paper also reported the same results [39]. In fact, the apparent contact angle of the P0 and P1 groups was considerable (31.78 ± 0.32 degrees). In contrast, the SBS of RelyX^TM^ U200 exhibited very little difference. It was assumed that the high SBS observed in the P2 group may have been influenced by the presence of argon gas, based on EDS analysis results. The ionized energy of the argon gas used for plasma irradiation is approximately 15.8 eV; the presence of this energy may have affected the bonding strength [55].

Zirconia is increasingly used for dental prostheses owing to its excellent physical properties, biocompatibility [56], and suitability for fabrication of dental implants [57,58]. However, zirconia prostheses produced in dental laboratories can introduce a variety of infectious agents into the oral cavity [59]. In addition, zirconia implants are vulnerable to bacterial infections, because the implant surface is directly osseointegrated with alveolar bone, such that alveolar bone loss can occur rapidly if peri-implantitis occurs [60,61]. Generally, bacterial attachment to hydrophobic, nonpolar surfaces is more extensive than to hydrophilic surfaces [62]; thus, hydrophobic zirconia may be more susceptible to infections. Although mechanical and chemical methods for bacteria removal have been proposed to address this problem [63,64,65], physical removal is not practical, because it would require the removal of the pre-setting zirconia prosthesis or pre-installed implant from the oral cavity. Chemical removal can be hazardous because of the possibility of damage to the surrounding tissues or residual materials. Non-thermal plasma carries no risk associated with residual materials, as it does not leave any byproducts [66,67]; in addition, its beneficial antimicrobial effects have been reported [68,69]. 

The antibacterial effect of plasma is mainly due to the generation of reactive species. Plasmas derived from single or mixed gases convert atmospheric oxygen and nitrogen into reactive materials, such as ozone, nitric oxide, and hydroxyl radicals [70]. In this experiment, the increase in the levels of reactive oxygen species can be indirectly estimated through the detection of oxygen via XPS. These reactive oxygen species are considered to be a key component of cell inactivation, and can cause loss of cell membrane function and changes in gene expression [71]. Deng et al. found changes in the integrity of the cell membrane after plasma treatment, via fluorescence microscopy [72]. In addition, the plasma-induced reactive oxygen species increased the expression of the oxidative stress response genes, which in turn upregulated 18 genes associated with the SOS response, the first response to DNA damage. This SOS response interferes with the activation of the DNA repair systems in bacteria [73]. It is also known that reactive oxygen species affect electrostatic forces among various factors in the initial attachment effect. This force is the most important force when the distance between bacteria is 10–20 nm, and it is assumed that such a change in force is due to plasma, which may interfere with bacterial adhesion [74]. Decrease in carbon levels also affects bacterial adhesion. Decreases in carbon levels have been reported to have a negative effect on biological activity, which in turn reduces bacterial motility [42,75]. It is explained that these various plasma-induced chemical changes work in combination to reduce bacterial adhesion. Another factor associated with bacterial adhesion is the difference in the roughness of the material surface [76]. Many previous studies have suggested that changes in the roughness of the implant surface can affect bacterial adhesion [76,77]. However, no change in roughness was observed in this experiment using plasma, which proved that chemical changes alone, with no change in roughness, can inhibit bacterial adhesion. A few studies claim that chemical changes are more important than changes in roughness [78,79]. If using plasma, it would be better if a method that would generate the aforementioned set of chemical changes while making a smoother surface than plasma is used.

Although the effects of antibacterial agents have been reported previously, the duration of the antimicrobial effect of plasma treatment is unknown. In the present study, we determined the persistence of the antimicrobial effect of plasma treatment, and found that it lasted for approximately 48 h. In addition, the biofilm thickness of the experimental group, when measured immediately after plasma treatment, was significantly less than that of the experimental groups measured at 24 h and 48 h after plasma treatment. These data suggest that the greatest effect occurred immediately after treatment, even though the effect of the plasma treatment lasted for two days. The manner in which plasma affected the adhesion of *S. mutans* was revealed by the marked decrease in the apparent contact angle immediately after plasma treatment. This increased hydrophilicity has been suggested previously [80]. In this experiment, the presence of argon gas detected in the EDS analysis may also have influenced the persistence of the antimicrobial effect for two days, which was also consistent with prior findings [30]. However, given that plasma made from an argon/oxygen mixture is eight times more effective than that made from argon alone [81], further studies will be needed to determine the antimicrobial properties of plasmas derived from different gases. In addition, additional studies on complex factors due to hydrophilic surface, increase of surface energy, decarbonisation and other elements should be carried out.

This was a preliminary investigation of the bonding strength and antimicrobial effects of plasma treatment of zirconia, including when using MDP-containing resins [82], and thus the study was limited by the use of only one type of gas, time setting and a few specimens. It will be necessary to assess the use of various gases, different plasma treatment times, currents, and voltages, to optimize the effect on bonding strength and antibacterial properties. Further research is necessary to develop plasma that has a long-lasting effect in clinical situations.

## 5. Conclusions

The elapsed time after treatment with non-thermal atmospheric plasma affected the SBS between zirconia and resin cement, which varied depending on the type of cement. In addition, the antimicrobial effect of the plasma on *S. mutans* was greatest immediately after treatment, but persisted for some time afterward.

## Figures and Tables

**Table 1:** Plasma treatment and storage conditions in each group.

Group	Plasma Treatment	Storage Conditions
Control	No	-
P0	Yes	Immediate
P1	Yes	In atmosphere for 24 h
P2	Yes	In atmosphere for 48 h
P3	Yes	In atmosphere for 72 h

**Table 2:** Resin cements used in the present study.

Resin Cement	Type and Primary Composition	Manufacturer
RelyX U200^TM^	Self-cured (vitreous powder, methacrylate monomers, silanated fillers, initiator components)	3M ESPE
Panavia F2.0	Dual-cured (BPEDMA, 10-MDP, DMA, silica, initiators)	Kuraray Medical Inc.

Information provided by the manufacturer: BPEDMA, bisphenol-A-polyethoxy dimethacrylate; DMA, aliphatic dimethacrylate; 10-MDP, 10-methacryloxydecyl dihydrogen phosphate.

**Table 3:** Energy-dispersive X-ray spectroscopy elemental analysis after plasma treatment, according to different time intervals.

Group Element	Control		P0		P1		P2		P3	
Element	Weight (%)	Atomic (%)	Weight (%)	Atomic (%)	Weight (%)	Atomic (%)	Weight (%)	Atomic (%)	Weight (%)	Atomic (%)
O	28.01	50.54	50.56	71.40	37.40	77.30	30.39	70.61	32.09	60.49
Zr	58.18	18.09	69.15	28.33	62.58	22.68	69.16	28.19	58.46	19.33
C	12.93	31.08	-	-	-	-	0.36	1.12	7.98	20.03
Ar	-	-	0.29	0.27	0.02	0.02	0.09	0.08	-	-

Note: The sum of the four elements (C, carbon; O, oxygen; Ar, argon; Zr, zirconium) was assumed to be the total.

**Table 4:** Carbon/oxygen proportion of zirconia after plasma treatment.

Element	Control	P0	P1	P2	P3
Carbon (%)	61.57	23.94	30.46	28.62	37.30
Oxygen (%)	38.43	76.06	69.54	71.38	62.70
C/O proportion	1.60	0.31	0.44	0.40	0.59

C, carbon; O, oxygen.

**Table 5:** Shear bond strength (SBS) value and failure pattern analysis.

Group	SBS (MPa)	Failure Pattern
Panavia F2.0, Control	6.53 (0.81) ^a^	100% mixed
Panavia F2.0, P0	4.22 (0.99) ^b^	100% mixed
Panavia F2.0, P1	5.19 (0.92) ^a^	100% mixed
Panavia F2.0, P2	6.05 (1.07) ^a^	100% mixed
Panavia F2.0, P3	6.98 (1.69) ^a^	100% mixed
RelyX^TM^ U200, Control	3.08 (1.54) ^c^	10% mixed, 90% adhesive
RelyX^TM^ U200, P0	5.26 (0.83) ^d^	100% mixed
RelyX^TM^ U200, P1	5.08 (1.15) ^d^	100% mixed
RelyX^TM^ U200, P2	4.24 (0.76) ^d^	100% mixed
RelyX^TM^ U200, P3	2.71 (1.19) ^c^	100% adhesive

Note: Groups with the same superscript were not significantly different.
